# Teaching Ordinal Patterns to a Computer: Efficient Encoding Algorithms Based on the Lehmer Code

**DOI:** 10.3390/e21101023

**Published:** 2019-10-21

**Authors:** Sebastian Berger, Andrii Kravtsiv, Gerhard Schneider, Denis Jordan

**Affiliations:** 1 Department of Anaesthesiology and Intensive Care, Klinikum rechts der Isar der Technischen Universität München, 81675 Munich, Germany; andrii.kravtsiv@tum.de (A.K.); gerhard.schneider@tum.de (G.S.); 2 Institute of Geomatics Engineering, University of Applied Sciences and Arts Northwestern Switzerland, 4132 Muttenz, Switzerland; denis.jordan@fhnw.ch

**Keywords:** Lehmer code, ordinal patterns, symbolic dynamics, permutation entropy, symbolic transfer entropy

## Abstract

Ordinal patterns are the common basis of various techniques used in the study of dynamical systems and nonlinear time series analysis. The present article focusses on the computational problem of turning time series into sequences of ordinal patterns. In a first step, a numerical encoding scheme for ordinal patterns is proposed. Utilising the classical Lehmer code, it enumerates ordinal patterns by consecutive non-negative integers, starting from zero. This compact representation considerably simplifies working with ordinal patterns in the digital domain. Subsequently, three algorithms for the efficient extraction of ordinal patterns from time series are discussed, including previously published approaches that can be adapted to the Lehmer code. The respective strengths and weaknesses of those algorithms are discussed, and further substantiated by benchmark results. One of the algorithms stands out in terms of scalability: its run-time increases linearly with both the pattern order and the sequence length, while its memory footprint is practically negligible. These properties enable the study of high-dimensional pattern spaces at low computational cost. In summary, the tools described herein may improve the efficiency of virtually any ordinal pattern-based analysis method, among them quantitative measures like permutation entropy and symbolic transfer entropy, but also techniques like forbidden pattern identification. Moreover, the concepts presented may allow for putting ideas into practice that up to now had been hindered by computational burden. To enable smooth evaluation, a function library written in the C programming language, as well as language bindings and native implementations for various numerical computation environments are provided in the supplements.

## 1. Introduction

The article *Permutation Entropy: A Natural Complexity Measure for Time Series* by Christoph Bandt and Bernd Pompe [1] pioneered a novel approach towards nonlinear time series analysis. In essence, the time series of interest is embedded in an *m*-dimensional phase space, then each delay vector is discretised according to the ordinal ranks among its *m* components. This procedure yields a sequence of symbols synonymously called order patterns or ordinal patterns, whereas the parameter *m* is either referred to as the embedding dimension, or simply the *order* of the ordinal pattern. Permutation entropy (PeEn) is in turn defined as the Shannon entropy [2,3] of a marginal probability distribution of such ordinal patterns.

Comprehensive overviews on the many applications of PeEn are given in [4,5,6]. Following the initial publication of 2002 [1], numerous extensions and modifications of PeEn have been devised, for instance, the methods proposed in [7,8,9,10]. Apart from Shannon entropy, other information-theoretic measures have been applied to ordinal pattern distributions, among them conditional entropy [11], mutual information [12], and transfer entropy [13,14]. Recurrence plots [15] and various correlation functions [16] were also transferred to the so-called ordinal pattern space [17]. On a more abstract level, ordinal patterns were tightly integrated into the general theory of discrete dynamics. A thorough introduction to such topics is provided in the book *Permutation Complexity in Dynamical Systems* by José Amigó [18].

### 1.1. Efficient but Infeasible?

Besides its conceptual simplicity and its robustness against certain forms of measurement noise, computational efficiency is likely one of the most-cited advantages of the Bandt–Pompe approach towards time series analysis [1,4,6,7,8,9,10,11,14,15,19,20,21,22,23,24]. However, this well-acclaimed run-time behaviour is not a specific property of ordinal analysis, but constitutes a feature of discrete dynamics in general. By matter of principle, coarse-graining the phase space of a dynamical system can radically reduce the computational cost of its analysis because quantisation turns continuous probability densities into discrete probability masses. A concise example (intentionally unrelated to ordinal patterns) can be found in [25], wherein Andreas Kaiser and Thomas Schreiber address the intricacies of estimating transfer entropy from continuously-valued time series, as compared to the far simpler discrete case.

Before the computational benefits of symbolisation can take any effect in ordinal analysis, the (usually real-valued) input data need to be converted into sequences of discrete ordinal patterns. Somewhat paradoxically, extracting ordinal patterns from time series is computationally a lot heavier than literature commonly suggests. Determining a single ordinal pattern of order *m* requires a total of $(m^{2}-m)/2$ pairwise comparisons, resulting in a computational complexity of $O(m^{2})$. In other words, “the computation time increases rapidly with *m*”—as has been pointed out by Matthäus Staniek and Klaus Lehnertz [26], the creators of symbolic transfer entropy [14]. In a similar context, Amigó stated that “there is no substitute for substantial computational effort when [the order *m*] becomes sufficiently large”, and further conjectured that working with ordinal patterns beyond approximately m=12 may likely be “computationally unfeasible” [18].

Besides computational complexity, another closely related issue is the spatial complexity of ordinal analysis: how should ordinal patterns best be represented in the digital domain, and what amount of extra memory is required to obtain that representation? Because a total of m! different ordinal patterns of order *m* exist (see Section 2.2), their memory footprint scales at a super-exponential rate of O(m!). Although posing a computational challenge to the investigator, this combinatorial explosion also has a beautiful application in the study of complex dynamics. The increasing spatial complexity of ordinal patterns will at some point transcend the irregularity producible by any chaotic dynamics, which gives rise to the notion of so-called forbidden ordinal patterns. This term describes patterns that do exist in theory, but cannot be generated under the particular evolution rule of a given dynamical system. Their presence or absence can therefore guide the distinction between complex determinism and mere randomness [18,27,28]. In the words of Amigó and colleagues: “Chaos […] cannot cope with a super-exponentially growing manifold such as that of order patterns” [27].

Against this backdrop, and especially considering the semantic gap between “high efficiency” and downright “infeasibility”, it seems justified to elaborate on the computational pitfalls and algorithmic possibilities of encoding ordinal patterns. Literature on this subject is still rather scarce. To the best of our knowledge, there is only one group of researchers who have published on the topic until now, which is the team led by Karsten Keller. In their white paper on ordinal analysis, Karsten Keller, Mathieu Sinn and Jan Emonds proposed a numerical encoding scheme for ordinal patterns [17], which Valentina Unakafova and Karsten Keller subsequently optimised for speed of execution [21]. The results of their work enable the efficient extraction of ordinal patterns from time series for the most commonly used pattern orders [29] that is, for m∈{2,3,…,9}. (To avoid ambiguity, please note that the Keller group prefers a different definition of the pattern order, according to which the ordinal pattern of an *m*-dimensional vector is of order d=m−1.) Expanding on the aforementioned work of the Keller group [17,21], the present article proposes to use the Lehmer code for mapping ordinal patterns onto non-negative integer values, and demonstrates that simple, efficient and versatile algorithms result from this modification.

### 1.2. Structure of the Article

Section 2 discusses a few simple (yet rather uncommon) mathematical concepts and notations, including the definition of ordinal patterns used throughout the present article. On that basis, Section 3 discusses the Lehmer code as an advantageous numerical representation for ordinal patterns, and presents a closed-form solution for their encoding. A comparable approach, originally proposed in [17], is also summarised and put into context. Section 4 then expands on these concepts, describing three different algorithms for transforming univariate time series into sequences of ordinal patterns—among them, the aforementioned solution by Unakafova and Keller [21]. The main computational challenges of turning those algorithms into run-time efficient code are addressed in Section 5, and possible optimisation techniques are suggested. Both aspects are immediately substantiated by run-time measurements. The hurried reader can safely skip to the concluding Section 6, which should provide enough information to put the ideas of the article into action by utilising the supplementary software library. That being said, readers concerned with the inner workings of ordinal pattern encoding (and those who lack blind trust in foreign code) are invited to follow through the article in its entirety. Let us start with some mathematical underpinnings.

## 2. Preliminaries

### 2.1. Iversonian Brackets

Throughout this article, we will be using a highly convenient, if slightly uncommon notational convention, called the Iversonian bracket [30,31]. In terms of computer science, the Iversonian bracket represents a data type conversion from Boolean to integer: for a given logical expression *L*, it holds that

(1) $$\left[L\right]=\left\{\begin{array}{cc} 0, & \mathrm{if}\;L\;\mathrm{is}\;\mathrm{false},\\ 1, & \mathrm{if}\;L\;\mathrm{is}\;\mathrm{true}. \end{array}\right.$$

For example, the number of positive elements in a finite time series $\left\{x_{1},x_{2},\ldots ,x_{N}\right\}$ can compactly be written as $\sum_{t=1}^{N}\left[x_{t}>0\right]$.

### 2.2. Ordinal Patterns

Any *m*-tuple $\left(x_{1},x_{2},\ldots ,x_{m}\right)\in X^{m}$ of pairwise distinct elements from a totally ordered set *X* has a unique ordinal pattern. This abstract entity describes how the tuple’s elements relate to one another in terms of position and rank order. For example, the ordinal pattern of the tuple $\left(17,7,8\right)\in N^{3}$ is fully specified by:

(2) “Therearethreeelements,thefirstisthegreatest,thesecondistheleast.”

This same ordinal pattern applies to any tuple $\left(x_{1},x_{2},x_{3}\right)$ for which the order relations $x_{2}<x_{3}<x_{1}$ hold. By contrast, each permutation of the elements $\left\{x_{1},x_{2},x_{3}\right\}$ yields a different ordinal pattern, so any given *m*-tuple of pairwise distinct elements $\left(x_{1},x_{2},\ldots ,x_{m}\right)\in X^{m}$ has exactly one out of m! different ordinal patterns. We here call the tuple length m∈{2,3,…} the order of the set of ordinal patterns $\Omega_{m}=\left\{\Pi_{1},\Pi_{2},\ldots ,\Pi_{m!}\right\}$. Any such pattern $\Pi_{i}\in \Omega_{m}$ can formally be denoted by a distinct permutation function

$$\sigma :N\to N,\;\mathrm{such}\;\mathrm{that}\;x_{\sigma (1)}<x_{\sigma (2)}<\ldots <x_{\sigma (m)},$$

or, equivalently, by its inverse function

$$\sigma^{-1}:N\to N,\;\mathrm{such}\;\mathrm{that}\;\sigma^{-1}\left(i\right)<\sigma^{-1}\left(j\right)\Leftrightarrow x_{i}<x_{j}.$$

Intuitively, the permutation function sorts the tuple, whereas its inverse function assigns a unique rank to each element. Variants of both notations coexist in literature [1,14,16,17,18,23]. For the scope of the current article, we choose to represent ordinal patterns as *m*-tuples of ranks $\left(\lambda_{1},\lambda_{2},\ldots ,\lambda_{m}\right)\in N^{m}$, where $\lambda_{i}=\sigma^{-1}\left(i\right)$.

The strict limitation to pairwise distinct elements is usually dropped in practise. The common approach is to stipulate $\lambda_{i}<\lambda_{j}$ for any pair of values $x_{i}=x_{j}$ if their order of appearance is i<j, and vice versa. (Depending on the amplitude distribution of the input data [22], it may be advisable to use a more sophisticated technique of resolving tied values during data pre-processing, that is, prior to the rank analysis considered here.) Adopting this simple convention, we arrive at the following definition.

**Definition** **1.** *For any given m-tuple $\left(x_{1},x_{2},\ldots ,x_{m}\right)\in X^{m}$ from a totally ordered set X, its ordinal pattern $\Pi_{i}\in \Omega_{m}=\left\{\Pi_{1},\Pi_{2},\ldots ,\Pi_{m!}\right\}$ of order m is the unique m-tuple of ranks $\left(\lambda_{1},\lambda_{2},\ldots ,\lambda_{m}\right)\in {\left\{1,2,\ldots ,m\right\}}^{m}$, such that*

(3) $$\forall i,j\in \left\{1,2,\ldots ,m\right\}:\;\lambda_{i}<\lambda_{j}\Leftrightarrow \left(x_{i}<x_{j}\;\vee \;\left(x_{i}=x_{j}\;\wedge \;i<j\right)\right).$$

Note that, under this definition, any given $\Pi_{i}\in \Omega_{m}$ does not only represent an ordinal pattern, but rather *is* the ordinal pattern itself. To motivate this, let us further introduce the function

(4) $$\begin{array}{cc} \mathrm{op}:X^{m} & \to \Omega_{m}\subset N^{m},\\ \left(x_{1},x_{2},\ldots ,x_{m}\right) & \mapsto (\lambda_{1},\lambda_{2},\ldots ,\lambda_{m}), \end{array}$$

which enables statements like $\Pi_{i}=\mathrm{op}\left(x_{1},x_{2},\ldots ,x_{m}\right)$. Then, due to Definition 1, the rather curious expression $\mathrm{op}(\Pi_{i})$ is actually well-defined: it is the ordinal pattern of the ordinal pattern $\Pi_{i}$, which simply is the ordinal pattern of an *m*-tuple of pairwise distinct positive integers. Moreover, it is easily confirmed that $\mathrm{op}\left(\Pi_{i}\right)=\Pi_{i}$ for all $\Pi_{i}\in \Omega_{m}$, which implies that the function op=op∘op is an idempotence—the ordinal pattern of another ordinal pattern is that other ordinal pattern.

### 2.3. Ordinal Processes and Markov Chains

The fundamental idea presented by Bandt and Pompe is to transform a given time series $\left\{x_{t}\right\}$ with time indices t∈{1,2,…} into a sequence of discrete symbols $\left\{\Pi_{t}\right\}$ prior to any further processing. This approach builds upon the delay embeddings used in dynamical systems theory. Using a fixed pattern order m∈{2,3,…} and a time lag τ∈{1,2,…}, one creates the sequence

(5) $$\left\{\Pi_{t}\right\}\;\mathrm{with}\;\Pi_{t}=\mathrm{op}\left(x_{t},x_{t+\tau },\ldots ,x_{t+(m-1)\tau }\right),$$

that is, the ordinal patterns of the consecutive delay vectors of $\left\{x_{t}\right\}$. In the light of Definition 1, this transformation combines delay embeddings with a nonlinear form of vector quantisation. For this reason, some authors prefer to call the pattern order *m* the embedding dimension of the ordinal pattern.

Assuming that the time series $\left\{x_{t}\right\}$ is a realisation of some stochastic process, it makes sense to postulate that its corresponding sequence of ordinal patterns $\left\{\Pi_{t}\right\}$ originates from an underlying stochastic process as well. In particular, this process is time-discrete, and its state space is the set of ordinal patterns $\Omega_{m}$. Keller, Sinn, and Emonds coined the term ordinal process for this concept [17]. The most decisive property of an ordinal process of order m>2 is that its random variables can never be independent. In this respect, observe that, for any time *t*, the *m*-dimensional delay vectors

$$\left(x_{t},x_{t+\tau },\ldots ,x_{t+(m-1)\tau }\right)\;\mathrm{and}\;\left(x_{t+\tau },x_{t+2\tau },\ldots ,x_{t+m\tau }\right)$$

overlap in m−1 out of *m* values. Consequently, with $\Pi_{t}$ already fixed, an ordinal process cannot draw $\Pi_{t+\tau }$ from its full state space $\Omega_{m}$, but merely from a subset of cardinality m!/(m−1)!=m. Keller and colleagues regard this property as the very definition [17] of ordinal processes, by contrast with any other process drawing from the state space $\Omega_{m}$. As already mentioned, ordinal processes of order m=2 constitute an exception in this regard because $\left(x_{t},x_{t+\tau }\right)$ and $\left(x_{t+\tau },x_{t+2\tau }\right)$ overlap in merely one value, such that their ordinal patterns have disjoint order relations. Consistently, it holds that 2!=2.

For the time lag τ=1, an ordinal process is a first-order Markov chain. Because of the inter-pattern dependencies just described, its corresponding transition matrix T is sparse, containing no more than *m* positive entries per row. Assuming m=3, for instance, the matrix cannot be less sparse than

$$T=\begin{array}{cc} & \begin{array}{cccccc} \left(1,2,3\right) & \left(1,3,2\right) & \left(2,1,3\right) & \left(2,3,1\right) & \left(3,1,2\right) & \left(3,2,1\right) \end{array}\\ \begin{array}{c} \left(1,2,3\right)\\ \left(1,3,2\right)\\ \left(2,1,3\right)\\ \left(2,3,1\right)\\ \left(3,1,2\right)\\ \left(3,2,1\right) \end{array} & \left(\begin{array}{cccccc} \;p_{1,1}\; & \;p_{1,2}\; & \;0\; & \;p_{1,4}\; & \;0\; & \;0\;\\ \;0\; & \;0\; & \;p_{2,3}\; & \;0\; & \;p_{2,5}\; & \;p_{2,6}\;\\ \;p_{3,1}\; & \;p_{3,2}\; & \;0\; & \;p_{3,4}\; & \;0\; & \;0\;\\ \;0\; & \;0\; & \;p_{4,3}\; & \;0\; & \;p_{4,5}\; & \;p_{4,6}\;\\ \;p_{5,1}\; & \;p_{5,2}\; & \;0\; & \;p_{5,4}\; & \;0\; & \;0\;\\ \;0\; & \;0\; & \;p_{6,3}\; & \;0\; & \;p_{6,5}\; & \;p_{6,6}\; \end{array}\right) \end{array}.$$

**Figure 1:**
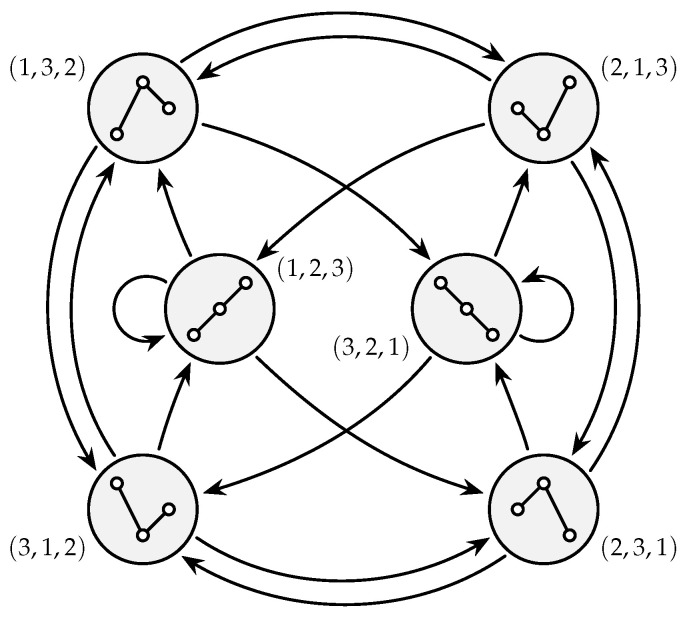
State diagram for an ordinal process of order m=3 and time lag τ=1, which can be interpreted as a first-order Markov chain. Its transition probabilities depend on the underlying process, and some can be zero. However, no other transitions than the ones depicted are possible because consecutive patterns overlap in two out of three values.

In the general case of τ>1, an ordinal process behaves like τ such Markov chains interleaved. By way of further illustration, a state diagram for the order m=3 (and necessarily, the time lag τ=1) is depicted in Figure 1.

## 3. Ordinal Patterns in the Digital Domain

In the last section, we defined ordinal patterns as *m*-tuples of ranks $\left(\lambda_{1},\lambda_{2},\ldots ,\lambda_{m}\right)\in \Omega_{m}\subset N^{m}$, and thereby also established an easily interpretable means of notation. Human interpretability is, however, not a primary concern when storing data in computer memory. Different requirements then prevail, and render the rank representation rather cumbersome. Before we discuss suitable encodings for the digital domain, let us look at these requirements.

Assume that we want to store an ordinal pattern $\left(\lambda_{1},\lambda_{2},\ldots ,\lambda_{m}\right)$ of order *m* in the main memory of a computer. The naïve solution (for any m<256) would then be to use an array of *m* consecutive bytes, each holding a particular rank $\lambda_{i}$. This approach is disadvantageous in several respects, the most prominent being:

1. While there are m! distinct ordinal patterns of order *m*, a block of *m* bytes can take on $256^{m}$ different states. Due to $m!\ll 256^{m}$ for small *m*, the memory footprint of the above encoding is far from optimal.
2. Testing a pair of ordinal patterns for equality requires up to *m* byte-wise comparisons, which is particularly detrimental to the run-time of operations like sorting and searching.
3. Counting distinct pattern occurrences in a sequence of ordinal patterns requires an associative array that provides a map from each possible tuple of ranks to its respective counter variable.

Those shortcomings can be overcome by representing the patterns $\Omega_{m}=\left\{\Pi_{1},\Pi_{2},\ldots ,\Pi_{m!}\right\}$ using non-negative integers {0,1,…,m!−1} that is, by establishing a bijective map,

(6) $$\begin{array}{cc} \mathrm{enc}:\Omega_{m} & \to N_{0},\\ \Pi_{i} & \mapsto i-1. \end{array}$$

Such a map could readily be implemented in software by means of a lookup table. However, encoding a pattern would then require up to m! iterations of that table, with each iteration involving up to *m* integer comparisons. Fortunately, literature knows better ways of enumerating permutations, and, thus, of encoding ordinal patterns numerically.

### 3.1. The Keller–Sinn–Emonds Code

Against this backdrop, Keller, Sinn, and Emonds [17] proposed a closed-form solution that directly maps a given *m*-tuple of values $\left(x_{1},x_{2},\ldots ,x_{m}\right)\in X^{m}$ onto its ordinal pattern in numerical representation, that is, the authors suggested a function of the form

$$\mathrm{enc}\circ \mathrm{op}=\mathrm{sym}:X^{m}\to N_{0}.$$

The principle is as follows: given an ordinal pattern $\Pi_{i}=\left(\lambda_{1},\lambda_{2},\ldots ,\lambda_{m}\right)\in \Omega_{m}$ of order *m*, one interprets its ranks $\left(\lambda_{1},\lambda_{2},\ldots ,\lambda_{m}\right)$ as a permutation of the integers {1,2,…,m}, and in turn obtains its right inversion counts

(7) $$\left(r_{1},r_{2},\ldots ,r_{m}\right)\;\mathrm{where}\;r_{i}=\sum_{j=i}^{m}\left[\lambda_{i}>\lambda_{j}\right].$$

For any fixed pattern order *m*, there are a total of m! pairwise distinct tuples $\left(r_{1},r_{2},\ldots ,r_{m}\right)$, and each corresponds to a particular ordinal pattern $\Pi_{i}\in \Omega_{m}$. Any such tuple of inversion counts can then bijectively be mapped onto a distinct non-negative integer

(8) $$n=\sum_{i=1}^{m}r_{i}\;\frac{m!}{(m-i+1)!}\;\mathrm{such}\;\mathrm{that}\;n\in \left\{0,1,\ldots ,m!-1\right\}.$$

With $r_{m}=0$ for the rightmost right inversion count, and $\lambda_{i}>\lambda_{j}$ if and only if $x_{i}>x_{j}$ (see Definition 1), the ordinal pattern symbolisation function

(9) $$\begin{array}{cc} {\mathrm{sym}}^{*}:X^{m} & \to N_{0},\\ \left(x_{1},x_{2},\ldots ,x_{m}\right) & \mapsto \sum_{i=1}^{m-1}\left(\frac{m!}{(m-i+1)!}\sum_{j=i+1}^{m}\left[x_{i}>x_{j}\right]\right) \end{array}$$

follows immediately. This function maps any *m*-tuple of values $\left(x_{1},x_{2},\ldots ,x_{m}\right)\in X^{m}$ onto a distinct numerical representation of its ordinal pattern. As discussed in the beginning of the current section, this is highly advantageous in computational terms. Still, a minor drawback of Equation (9) is that factorial functions and integer division operations are involved, which are computationally expensive in general. As suggested in [21], this can be resolved by computing the weights

$$w_{i}=\frac{m!}{(m-i+1)!}\;\mathrm{for}\;i\in \left\{1,2,\ldots ,m-1\right\}$$

only once in advance, and looking them up during the actual encoding. Alternatively, the encoding given by Equation (8) can be modified, as will be discussed in the following.

### 3.2. The Lehmer Code

Named in appreciation of Derrick Lehmer, the Lehmer code assigns a unique non-negative integer n∈{0,1,…,m!−1} to each permutation of a set of *m* elements. The mathematical foundations of this problem had already been studied in the 19th century [32], and Lehmer incorporated them into his work on algorithms for combinatorial computing, published as *Teaching Combinatorial Tricks to a Computer* [33] in 1960. Lehmer’s approach towards enumerating permutations is conceptually similar to the solution that Keller and colleagues [17] proposed for the encoding of ordinal patterns. Given some permutation $\left(\lambda_{1},\lambda_{2},\ldots ,\lambda_{m}\right)$, which could be the ranks of an ordinal pattern $\Pi_{i}\in \Omega_{m}$ without loss of generality, its corresponding set of right inversion counts $\left(r_{1},r_{2},\ldots ,r_{m}\right)$ in terms of Equation (7) are obtained. Then, and by contrast with Equation (8), a unique integer representation of that permutation is calculated according to

(10) $$n=\sum_{i=1}^{m}r_{i}\left(m-i\right)!\;\mathrm{such}\;\mathrm{that}\;n\in \left\{0,1,\ldots ,m!-1\right\}.$$

In other words, the tuple of right inversion counts $\left(r_{1},r_{2},\ldots ,r_{m}\right)$ is interpreted as an *m*-digit factoradic numeral of the form “$r_{1}r_{2}\ldots r_{m}$” [33]. In analogy with Section 3.1, but utilising the Lehmer code instead, the ordinal pattern of an *m*-tuple $\left(x_{1},x_{2},\ldots ,x_{m}\right)\in X^{m}$ can therefore be extracted and encoded by computing the function

(11) $$\begin{array}{cc} \mathrm{sym}:X^{m} & \to N_{0},\\ \left(x_{1},x_{2},\ldots ,x_{m}\right) & \mapsto \sum_{i=1}^{m-1}\left(\left(m-i\right)!\;\sum_{j=i+1}^{m}\left[x_{i}>x_{j}\right]\right). \end{array}$$

With a view to software implementation, Equation (11) still provides opportunity for optimisation. In its current form, the factorial (m−i)! needs to be re-evaluated for each iteration of the outer sum. In general, calculating a (non-trivial) factorial requires a sequence of multiplications, but, due to the specific structure of Equation (11), these multiplications are avoidable here in their entirety. Taking advantage of the fundamental recurrence relation k!=k(k−1)!, the value of $\mathrm{sym}(x_{1},x_{2},\ldots ,x_{m})$ can be computed recursively by initialising $n_{0}=0$, and successively iterating

(12) $$n_{i}=\left(m-i\right)\left(n_{i-1}+r_{i}\right)\;\mathrm{with}\;r_{i}=\sum_{j=i+1}^{m}\left[x_{i}>x_{j}\right].$$

The recursion terminates after iteration i=m−1, and yields $n_{m-1}=\mathrm{sym}\left(x_{1},x_{2},\ldots ,x_{m}\right)$ as the result. The arithmetical equivalence with Equation (11) can be proven by mathematical induction, and is also evident from the following example.

**Example** **1.** *Let $\left(r_{1},r_{2},\ldots ,r_{6}\right)\in R_{6}$ denote the right inversion counts to the ordinal pattern of a given tuple $\left(x_{1},x_{2},\ldots ,x_{6}\right)$. According to Equation* (11)*, the numerical representation of this ordinal pattern of order m=6 is obtained by computing*

$$\begin{array}{cccc} \mathrm{sym}(x_{1},x_{2},\ldots ,x_{6}) & = & 5\times 4\times 3\times 2\times r_{1}\\ \; & + & 4\times 3\times 2\times r_{2}\\ \; & + & 3\times 2\times r_{3}\\ \; & + & 2\times r_{4}\\ \; & + & r_{5} & \;. \end{array}$$

*Iterating the recurrence relation given by Equation* (12) *for the same right inversion counts $\left(r_{1},r_{2},\ldots ,r_{6}\right)$ and the same pattern order m=6, we obtain*

$$n_{5}\;=\;\mathrm{sym}\left(x_{1},x_{2},\ldots ,x_{6}\right)\;=\;\left(\left(\left(r_{1}\times 5\;+\;r_{2}\right)\times 4\;+\;r_{3}\right)\times 3\;+\;r_{4}\right)\times 2\;+\;r_{5}.$$

*Not only are both solutions arithmetically identical, but the recursive approach also requires considerably fewer multiplications—quod erat illustrandum.*

The recursion given by Equation (12) thus enables a remarkably simple algorithm for extracting and storing ordinal patterns in computer memory, as is demonstrated by the pseudocode of the following Algorithm 1.

**Algorithm 1.** Given an *m*-tuple $\left(x_{1},x_{2},\ldots ,x_{m}\right)\in X^{m}$ of elements from a totally ordered set *X*, a distinct numerical representation n∈{0,1,…,m!−1} of its ordinal pattern of order *m* can be obtained as outlined by the following pseudocode.1 **function** encode_pattern 2 **input** 3   $\left(x_{1},x_{2},\ldots ,x_{m}\right)\in X^{m}$
**with**
m∈{2,3,…} 4 **output** 5   *n*
∈{0,1,…,m!−1} 6 **begin** 7   n←0 8   **for**
i←1
**to**
m−1
**do** 9     **for**
j←i+1
**to**
*m*
**do** 10       $n\leftarrow n+\left[x_{i}>x_{j}\right]$ 11     **end** 12     n←(m−i)n 13   **end** 14   **return** n 15 **end.**

The computational complexity of Algorithm 1 in dependence of the pattern order *m* is $O(m^{2})$, as is further substantiated in Section 5.1.

Compared to the encoding scheme originally proposed in [17] and summarised in Section 3.1, adopting the Lehmer code allows for algorithms that can be implemented without relying on either factorial functions and division operations, or on lookup tables. Moreover, the encoding resulting from Equation (11) provides for an intuitive enumeration of ordinal patterns: figuratively speaking, the Lehmer code preserves the lexicographic sorting order of the permutations it encodes [33]. When applied to ordinal patterns, their tuples of ranks, tuples of inversion counts, as well as the resulting numerical codes are all consistently sorted. The reader may find Example 2 instructive in this respect.

**Example** **2.** *Consider the m!=24 ordinal patterns $\left(\lambda_{1},\lambda_{2},\lambda_{3},\lambda_{4}\right)\in \Omega_{4}$ of order m=4. Each of them has a distinct tuple of right inversion counts $\left(r_{1},r_{2},r_{3}\right)$, and its corresponding numerical representation n is obtained by interpreting $\left(r_{1},r_{2},r_{3}\right)$ as the digits of a factoradic numeral, in particular, $n=6r_{1}+2r_{2}+r_{3}$. As shown in Table 1, the tuples of ranks, tuples of inversion counts, and numerical codes all obey the same lexicographic sorting order.*

## 4. Encoding Time Series Data

Any form of ordinal pattern-based time series analysis requires that sequences of elements from a totally ordered set *X* (usually, the real numbers R) be converted into sequences of ordinal patterns. Given a finite time series $\left\{x_{t}\right\}$, with $x_{t}\in X$ and t∈{1,2,…,N}, we select a pattern order m⩾2 and time lag τ⩾1, and subsequently obtain $\left\{\Pi_{t}\right\}$, where

(13) $$\Pi_{t}=\mathrm{op}\left(x_{t},x_{t+\tau },\ldots ,x_{t+(m-1)\tau }\right)\;\mathrm{for}\;\mathrm{all}\;t\in \left\{1,2,\ldots ,N-\left(m-1\right)\tau \right\}.$$

In doing so, we assign to each ordinal pattern $\Pi_{t}$ the time index *t* of the leftmost of its underlying tuple elements. Consequently, the last (m−1)τ indices of the time series $\left\{x_{t}\right\}$ do not reference any ordinal pattern, and the resulting pattern sequence $\left\{\Pi_{t}\right\}$ thus comprises of N−(m−1)τ elements. To perform this transformation in software, the encoding approach described in the previous section can directly be utilised.

### 4.1. The Straightforward Approach (Plain Algorithm)

Algorithm 1 maps any given *m*-tuple $\left(x_{1},x_{2},\ldots ,x_{m}\right)\in X^{m}$ of elements from a totally ordered set *X* onto a non-negative integer n∈{0,1,…,m!−1}, such that the value

$$n\;=\;\mathrm{enc}\left(\mathrm{op}\left(x_{1},x_{2},\ldots ,x_{m}\right)\right)\;=\;\mathrm{sym}\left(x_{1},x_{2},\ldots ,x_{m}\right)$$

uniquely identifies the ordinal pattern $\mathrm{op}\left(x_{1},x_{2},\ldots ,x_{m}\right)=\left(\lambda_{1},\lambda_{2},\ldots ,\lambda_{m}\right)\in \Omega_{m}$ of the *m*-tuple. This encoding strategy is easily expanded, so as to turn an entire time series $\left\{x_{t}\right\}$ into numerical representations $\left\{n_{t}\right\}$ of its ordinal pattern sequence $\left\{\Pi_{t}\right\}$, whereby

(14) $$n_{t}=\mathrm{enc}\left(\Pi_{t}\right)=\mathrm{sym}\left(x_{t},x_{t+\tau },\ldots ,x_{t+(m-1)\tau }\right)\;\mathrm{for}\;\mathrm{all}\;t\in \left\{1,2,\ldots ,N-\left(m-1\right)\tau \right\}.$$

The extension of Algorithm 1 merely requires an additional loop and proper indexing. It results in the following algorithm.

In analogy with Algorithm 1, the computational complexity of Algorithm 2 is $O(m^{2})$ with regard to the pattern order *m*. It is O(N) in dependence of the sequence length *N*, and O(1) for any choice of the time lag τ (also see Section 5.1 and Section 5.8).

**Algorithm 2. Plain Algorithm.** To transform a finite time series $\left\{x_{t}\right\}$ of elements from a totally ordered set *X* into a sequence of non-negative integers $\left\{n_{t}\right\}$, select a pattern order m⩾2 and a time lag τ⩾1, and proceed according to the pseudocode below. The value $n_{t}\in \left\{0,1,\ldots ,m!-1\right\}$ then uniquely identifies the ordinal pattern $\Pi_{t}$ of order *m*, which is extracted from the time series $\left\{x_{t}\right\}$ at time index *t* using the time lag τ. The function encode_pattern is specified in Algorithm 1.
1 **function** encode_sequence 2 **input** 3  *m*
∈N
**with**
*m*
⩾2
4  τ
∈N
**with**
τ
⩾1 5  $\left\{x_{t}\right\}$ **with**
$x_{t}$
∈X
**and**
t∈{1,2,…,N} 6 **output** 7  $\left\{n_{t}\right\}$ **with**
$n_{t}$
∈{0,1,…,m!−1}
**and**
t∈{1,2,…,N−(m−1)τ} 8 **begin** 9  **for**
t←1
**to**
N−(m−1)τ
**do**
10    $n_{t}\leftarrow$ encode_pattern $\left(x_{t},x_{t+\tau },\ldots ,x_{t+(m-1)\tau }\right)$ 11    **end** 12 **end.**


### 4.2. An Optimised Encoding Strategy (Overlap Algorithm)

Algorithm 2 still contains some redundant operations, and does therefore not scale too well with the pattern order *m*. This aspect can be targeted by further optimisation. Bandt and Pompe had already hinted at this possibility in their seminal publication on ordinal patterns, suggesting there was “an extremely fast algorithm where each pair of values need to be compared only once” [1]. Keller, Sinn, and Emonds further elaborated on this matter, and demonstrated that the overlap property described in Section 2.2 can be exploited computationally [17]. The algorithm described in the following builds upon the same fundamental idea, but additionally uses the recursive Lehmer encoding proposed in Section 3.2.

Written out in its entirety, Algorithm 2 converts a time series $\left\{x_{t}\right\}$, indexed by t∈{1,2,…,N}, into a sequence of non-negative integers $\left\{n_{t}\right\}$, such that

(15) $$n_{t}=\sum_{i=1}^{m-1}\left(\left(m-i\right)!\;\sum_{j=i+1}^{m}\left[x_{t+(i-1)\tau }>x_{t+(j-1)\tau }\right]\right)\;\mathrm{for}\;\mathrm{all}\;t\in \left\{1,2,\ldots ,N-\left(m-1\right)\tau \right\}.$$

As derived in Section 3.2, each evaluation of the inner sum of Equation (15) yields one of the m−1 non-trivial right inversion counts to the ordinal pattern $\Pi_{t}$. The encoding can hence be reformulated in terms of

(16) $$n_{t}=\sum_{i=1}^{m-1}\left(m-i\right)!\times r_{t,\;i}\;\mathrm{where}\;r_{t,\;i}=\sum_{j=i+1}^{m}\left[x_{t+(i-1)\tau }>x_{t+(j-1)\tau }\right].$$

Now recall from Section 2.3 that any two ordinal patterns $\Pi_{t-\tau }$ and $\Pi_{t}$ at a distance equalling the time lag τ share all but one of their underlying time series values. Consequently, the inversion counts to the patterns $\Pi_{t-\tau }$ and $\Pi_{t}$ are strongly interrelated as well. In particular, it is easily confirmed that

(17) $$n_{t}=\sum_{i=1}^{m-1}\left(m-i\right)!\times r_{t,\;i}\;\mathrm{where}\;r_{t,\;i}=\left[x_{t+(i-1)\tau }>x_{t+(m-1)\tau }\right]\;+\;r_{t-\tau ,\;i+1}.$$

This recurrence relation is highly advantageous in computational terms. Let us assume that, in the overall process of encoding a time series $\left\{x_{t}\right\}$, the inversion counts to the pattern $\Pi_{t-\tau }$ have just been determined. If those are kept in memory for τ more iterations, then encoding the pattern $\Pi_{t}$ merely requires m−1 additional comparisons. In turn, each such comparison yields one of the inversion counts to the pattern $\Pi_{t}$, which can then be reused to efficiently encode $\Pi_{t+\tau }$ another τ time steps ahead. Merely the first τ ordinal patterns $\left\{\Pi_{1},\Pi_{2},\ldots ,\Pi_{\tau }\right\}$ require additional consideration because obviously, none of them has a neighbour located τ time steps earlier. While $\left\{\Pi_{1-\tau },\Pi_{2-\tau },\ldots ,\Pi_{0}\right\}$ are hence undefined, all but the leftmost of their respective inversion counts still formally exist. More precisely,

(18) $$R_{\mathrm{init}}\;=\;\left(\begin{array}{cccc} r_{1-\tau ,\;2} & r_{1-\tau ,\;3} & \ldots & r_{1-\tau ,\;m-1}\\ r_{2-\tau ,\;2} & r_{2-\tau ,\;3} & \ldots & r_{2-\tau ,\;m-1}\\ \vdots & \vdots & \ddots & \vdots \\ r_{0,\;2} & r_{0,\;3} & \ldots & r_{0,\;m-1} \end{array}\right)$$

are all well-defined, and suffice to calculate $\left\{n_{1},n_{2},\ldots ,n_{\tau }\right\}$ in terms of Equation (17). The procedure of encoding a time series thus comprises of two stages: (1) obtain the initial inversion counts $R_{\mathrm{init}}$, then (2) iterate Equation (17) for all *t*, starting from t=1. Each such iteration yields an encoded pattern $n_{t}$, as well as a corresponding set of inversion counts. The latter are temporarily buffered in memory, reused τ iterations later, and then become obsolete. The entire process is summarised in Algorithm 3.

**Algorithm 3. Overlap Algorithm.** To transform a finite time series $\left\{x_{t}\right\}$ of elements $x_{t}\in X$ into a sequence of non-negative integers $\left\{n_{t}\right\}$ representing the ordinal patterns $\left\{\Pi_{t}\right\}$ of the time series, select a pattern order m⩾2 and time lag τ⩾1. Then, proceed as follows.
1 **function** encode_sequence 2 **input** 3  $\left\{x_{t}\right\}$ **with**
$x_{t}$
∈X
**and**
t∈{1,2,…,N} 4  *m*
∈N
**with**
*m*
⩾2 5  τ
∈N
**with**
τ
⩾1 6 **output** 7  $\left\{n_{t}\right\}$ **with**
$n_{t}$
∈{0,1,…,m!−1}
**and**
t∈{1,2,…,N−(m−1)τ} 8 **locals** 9  $\left\{r_{i,\;j}\right\}$ **with**
i∈{1,2,…,τ}
**and**
j∈{1,2,…,m} 10 **begin** 11  $r_{i,\;j}\leftarrow 0$
**for all**
i∈{1,2,…,τ}
**and all**
j∈{1,2,…,m} 12 13  /* *Obtain initial right inversion counts *$R_{\mathrm{init}}$ */ 14  **for**
t←1
**to**
*τ*
**do** 15   **for**
i←1
**to**
m−2
**do** 16     **for**
j←i+1
**to**
m−1
**do** 17     $r_{t,\;i+1}\leftarrow r_{t,\;i+1}+\left[x_{t+(i-1)\tau }>x_{t+(j-1)\tau }\right]$ 18    **end** 19   **end** 20  **end** 21 22  /* *Extract and encode ordinal patterns* */ 23  i←1 24  **for**
t←1
**to**
N−(m−1)τ
**do** 25    **for**
j←1
**to**
m−1
**do** 26     $r_{i,\;j}$
$\leftarrow r_{i,\;j+1}+\left[x_{t+(j-1)\tau }>x_{t+(m-1)\tau }\right]$ 27    $n_{t}$
$\leftarrow \left(m-j\right)\left(n_{t}+r_{i,\;j}\right)$ 28    **end** 29   i←(imodτ)+1/* Increment circular buffer index */ 30  **end** 31 **end.**


Notice that we favour readability over efficiency in the pseudocode of Algorithm 3. Actually, a smaller buffer $\left\{r_{i,\;j}\right\}$ with i∈{1,…,τ} and j∈{2,…,m−1} will suffice for an actual implementation because the recurrence relation does not depend on $r_{i,\;1}$ at all, whereas $r_{i,\;m}=0$ always. In addition, the modulo operation used for circular buffer indexing may impose a considerable run-time penalty. Both aspects are addressed in the reference implementation of Algorithm 3, which is provided by the ordpat_encode_overlap function in the supplementary file ordpat.c.

Independent of such details of implementation, and let aside the initialisation of $R_{\mathrm{init}}$, the asymptotic computational complexity of Algorithm 3 is O(N) for the sequence length *N*, and a constant O(1) for the time lag parameter τ. By contrast with Algorithm 2, however, Algorithm 3 offers a complexity of O(m) with regard to the pattern order *m* (see Section 5.1 for details).

### 4.3. The Unakafova–Keller Approach (Lookup Algorithm)

**Figure 2:**
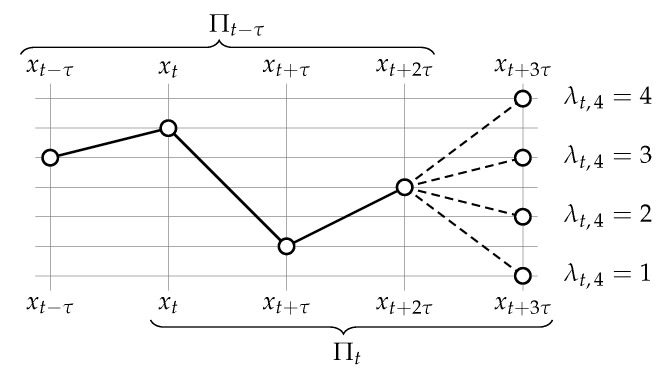
Assume pattern order m=4, without loss of generality. For any fixed ordinal pattern $\Pi_{t-\tau }$, its succeeding pattern $\Pi_{t}=\left(\lambda_{t,\;1},\lambda_{t,\;2},\lambda_{t,\;3},\lambda_{t,\;4}\right)$ at τ time steps distance has merely one degree of freedom: its rightmost rank $\lambda_{t,\;4}$.

Algorithm 3 is based on the fact that two ordinal patterns $\Pi_{t-\tau }$ and $\Pi_{t}$ overlap in all but one of their underlying time series values. Utilising the same interrelation, Valentina Unakafova and Karsten Keller proposed [21] a different encoding strategy that (by contrast with our Algorithm 3, as well as their own previous work [17]) does not depend on buffering inversion counts. The approach is compellingly simple: as described in Section 2.3, an ordinal pattern $\Pi_{t-\tau }$ of order *m* can only have *m* different succeeding patterns $\Pi_{t}$ at τ time steps distance. Consequently, if the ordinal pattern $\Pi_{t-\tau }=\mathrm{op}\left(x_{t-\tau },x_{t},\ldots ,x_{t+(m-2)\tau }\right)$ is known in advance, then the value of the expression

(19) $$\lambda_{t,\;m}=m-\sum_{i=1}^{m-1}\left[x_{t+(i-1)\tau }>x_{t+(m-1)\tau }\right]$$

uniquely determines the pattern $\Pi_{t}=\mathrm{op}\left(x_{t},x_{t+\tau },\ldots ,x_{t+(m-1)\tau }\right)$. In connection with Definition 1, the decisive variable $\lambda_{t,\;m}\in \left\{1,2,\ldots ,m\right\}$ is easily identified as the rightmost rank of the ordinal pattern $\Pi_{t}=\left(\lambda_{t,\;1},\lambda_{t,\;2},\ldots ,\lambda_{t,\;m}\right)$. As is visualised in Figure 2, each value that $\lambda_{t,\;m}$ can take on represents one of the *m* different ordinal patterns $\Pi_{t}$ that may possibly follow after a particular pattern $\Pi_{t-\tau }$.

These considerations essentially imply that a surjective map $\left(\Pi_{t-\tau },\;\lambda_{t,\;m}\right)\mapsto \Pi_{t}$ exists for any pattern order *m* and time lag τ. Likewise, there has to be a well-defined surjection

(20) $$\begin{array}{cc} N_{0}\times N & \to N_{0},\\ \left(n_{t-\tau },\;\lambda_{t,\;m}\right) & \mapsto n_{t} \end{array}$$

for ordinal patterns represented numerically. Note that the particular encoding function $\mathrm{enc}:\Omega_{m}\to N_{0}$ in terms of Equation (6) does not make a difference in this regard—as long as it is bijective, such that each ordinal pattern is assigned a unique numerical label. In their original publication [21], Unakafova and Keller used the encoding given by Equation (9). To implement Equation (20), the authors rely on a lookup table holding m!×m entries, in particular

(21) $$L_{m}=\left(\begin{array}{ccc} L_{1,\;1} & \ldots & L_{1,\;m}\\ \vdots & \ddots & \vdots \\ L_{m!,\;1} & \ldots & L_{m!,\;m} \end{array}\right)\;\mathrm{such}\;\mathrm{that}\;n_{t}=L_{i,\;j}\;\mathrm{for}\;i=n_{t-\tau }+1\;\mathrm{and}\;j=\lambda_{t,\;n}.$$

Encoding a time series $\left\{x_{t}\right\}$, with t∈{1,2,…,N}, is then a matter of computing the numerical codes $\left\{n_{1},n_{2},\ldots ,n_{\tau }\right\}$ for the first τ patterns by direct evaluation, and subsequently iterating Equation (20) to obtain the remaining symbols $\left\{n_{\tau +1},n_{\tau +2},\ldots ,n_{N-(m-1)\tau }\right\}$. Algorithm 4 describes the process in full detail.

**Algorithm 4.Lookup Algorithm.** To transform a finite time series $\left\{x_{t}\right\}$ into a sequence of non-negative integers $\left\{n_{t}\right\}$ representing its ordinal patterns, select a pattern order m⩾2 and time lag τ⩾1. In addition, prepare a lookup table $\left\{L_{i,\;j}\right\}$ according to Equation (21) that matches the encode_pattern function to be used. This can be the sym-function given by Equation (11), the ${\mathrm{sym}}^{*}$-function of Equation (9), or any other bijective map yielding $n_{t}\in \left\{0,1,\ldots ,m!-1\right\}$. Then, proceed as follows.1 **function** encode_sequence 2 **input** 3   $\left\{x_{t}\right\}$
**with**
$x_{t}$
∈X
**and**
t∈{1,2,…,N} 4   *m*
∈N
**with**
*m*
⩾2 5   τ
∈N
**with**
τ
⩾1 6 **output** 7   $\left\{n_{t}\right\}$
**with**
$n_{t}$
∈{0,1,…,m!−1}
**and**
*t*
∈{1,2,…,N−(m−1)τ} 8 **locals** 9   $\left\{L_{i,\;j}\right\}$
**with**
$L_{i,\;j}$
∈{0,1,…,m!−1}
**and**
*i*
∈{1,2,…,m!}
**and**
j∈{1,2,…,m} 10 **begin** 11   $\left\{L_{i,\;j}\right\}\leftarrow \text{load\_lookup\_table}\left(m\right)$ 12 13   /* Encode first τ ordinal patterns */ 14   **for**
t←1
**to**
*τ*
**do** 15     $n_{t}\leftarrow$
$\text{encode\_pattern}(x_{t},x_{t+\tau },\ldots ,x_{t+(m-1)\tau })$ 16   **end** 17 18   /* Encode all remaining patterns */ 19   **for**
t←τ+1
**to**
N−(m−1)τ
**do** 24     row
$\leftarrow n_{t-\tau }+1$ 20     col
←1 21     **for**
i←1
**to**
m−1
**do** 22      $col\leftarrow col+\left[x_{t+(i-1)\tau }>x_{t+(m-1)\tau }\right]$ 23     **end** 25     $n_{t}\leftarrow L_{row,\;col}$ 26   **end** 27 **end.**

In terms of computational complexity, the asymptotic behaviour of Algorithm 4 is identical to that of Algorithm 3. Putting aside the initialisation steps, its computational complexity is O(N) for the sequence length *N*, O(m) for the pattern order *m*, and O(1) with regard to the time lag τ. In practice, however, the run-time properties of the two algorithms can differ substantially. On the one hand, Algorithm 4 requires significantly fewer computational operations per ordinal pattern (see Table 2), as is the very purpose of using a lookup table. On the other hand, this reduction in computational complexity does come at a price—as will be further discussed in Section 5.7.

## 5. Implementation and Run-Time Performance

Three encoding algorithms of varying complexity have been presented in the previous section. Those will henceforth also be referred to by the plain algorithm (Algorithm 2), the overlap algorithm (Algorithm 3), and the lookup algorithm (Algorithm 4). Each algorithm comes with strengths and weaknesses, and raises different implementational challenges. Thus, the following section is intended to guide the reader in selecting and implementing the most appropriate algorithm for a particular execution platform and task. In particular, we will consider GNU Octave [34], Matlab (The Mathworks, Natick, MA, USA), NumPy/Python [35], and the C programming language.

### 5.1. Theoretical Computational Complexities

The computational complexities of the plain, overlap, and lookup algorithm (Algorithms 2–4) differ substantially in dependence of the pattern order *m*. This can be derived from the basic operation counts provided in Table 2.

Following from the total operation counts given in Table 2, the plain algorithm has an asymptotic computational complexity of $O(m^{2})$. By contrast, the overlap and lookup algorithm both scale linearly with the pattern order, that is, their complexity is O(m). However, the lookup algorithm avoids many of the computations that the overlap algorithm has to perform. Therefore, the theoretical computational complexity of the three algorithms decreases from the plain algorithm to the overlap algorithm, and again from the overlap algorithm to the lookup algorithm.

In practice, however, the way that an abstract algorithm is translated into actual software can make a big difference for its run-time efficiency. Taking into account application-dependent requirements and platform-specific peculiarities is essential in this regard. Therefore, the rest of this section focusses on those practical aspects of implementing the three encoding algorithms.

### 5.2. Memory Alignment

The algorithms described in Section 4 represent the ordinal patterns $\Omega_{m}=\left\{\Pi_{1},\Pi_{2},\ldots ,\Pi_{m!}\right\}$ by distinct integers {0,1,…,m!−1}, thereby providing a bijective map $\Pi_{i}\mapsto i-1$ as stipulated by the enc-function of Equation (6). The resulting symbols are highly memory-efficient, theoretically requiring a mere $\log_{2}m!$  per pattern. Note that $\log_{2}m!$ also is the entropy of a uniform distribution of m! elements, and, thus, the maximum entropy an ordinal pattern distribution of order *m* can possibly attain. Putting aside data compression techniques, no other encoding can be more compact (as is assured by Shannon’s source coding theorem [2,3]).

In practice, it makes sense to align ordinal patterns to byte boundaries, which can be accomplished by dedicating an integer power of 2 (but at least 8) bits to each ordinal pattern, such that the resulting bit width per pattern is

(22) $$w_{b}=2^{k}⩾\log_{2}m!\;\mathrm{where}\;k\in \left\{3,4,\ldots \right\}.$$

Any digital processor equipped with 64- integer registers will therefore handle ordinal patterns of order m⩽20 natively—that is, at hardware efficiency. For reference, Table 3 lists the maximum pattern orders that fit the primitive data types available on standard computer systems.

Although ordinal patterns and their numerical representations are intrinsically integral, Table 3 also references two IEEE 754 floating point formats [36,37]. Those were included because computation environments like NumPy/Python, GNU Octave, and Matlab by default use the binary64 floating point data format. Previously known as double [36], this format features an effective mantissa length of 53 [37], and can therefore represent a total of $2^{53}$ distinct non-negative values at integer precision [38]. If this limit is exceeded, mantissa truncation will silently cause unexpected results (like the erroneous $2^{53}=2^{53}+1$), and distinct patterns will falsely be labelled as identical. When working with patterns of order m>18 in such computation environments, this bug-inviting peculiarity must be kept in mind.

Independent of the software platform used, ordinal patterns of order m>20 require additional thought because current general-purpose processors do not provide native support for integers wider than 64 bits. Therefore, each pattern of order m>20 has to be stored as an array of integers, and all arithmetical and logical operations need to be emulated in software. Those matters will be further discussed in Section 5.6.

### 5.3. Run-Time Test Environment

All performance testing was done on a conventional x86-64 laptop computer, equipped with an Intel Core i7-5600U processor (Intel Corporation, Santa Clara, CA, USA) and 8G of random access memory (RAM). An Arch Linux distribution of the GNU/Linux operating system was used, running the default kernel (linux, 5.2.arch2-1) and C standard library (glibc, 2.29-3). Pre-built binary packages of GNU Octave (octave, 5.1.0-4), Python 3 (python, 3.7.3-1), NumPy (python-numpy, 1.16.4-1), and FFTW (fftw, 3.3.8-1), as well as their respective dependencies were installed from the official repositories of the distributor. The Linux version of the Matlab 2018b release was used. All source code written in the C programming language was compiled using the GNU Compiler Collection (gcc, 8.3.0-2). The parameters -march=x86-64 -mtune=generic -O3 were selected to allow for heavy compiler optimisation, while not relying on any model-specific features of the targeted processor.

### 5.4. Test Signal Generation

Based on the fact that ordinal patterns of any order are uniformly distributed in white noise [18], sequences of independent and uniformly distributed pseudo-random numbers were used to test the performance of the algorithms. This choice ascertains that all ordinal pattern transitions (see Figure 1) appear at the same relative frequency, such that each possible path of execution is taken equally often. In addition, to test for a possible dependency between the run-time of the algorithms and the ordinal complexity of the input signal, low-pass filtered (and thus, self-correlated) noise of various bandwidths was incorporated into the test procedure where appropriate. Filtering was performed by zeroing bins in the Discrete Fourier Transform (DFT) of the signal.

To maintain reproducible test signals across all software environments, the xorshift random number generator by George Marsaglia [39] was used with a word size of 32 bits and the standard shift parameters (13,17,5). In this configuration, xorshifting produces a pseudo-random sequence of period length $2^{32}-1$, and elements drawn from $\left\{1,2,\ldots ,2^{32}-1\right\}$. Normalisation (as in zero-mean or unit-variance) was omitted, considering that ordinal patterns are invariant to order-preserving transformations anyway [1]. However, the integer-valued test signals were stored in binary64 floating point representation, as this is the expected input format in ordinal pattern analysis.

### 5.5. The Plain Algorithm (Algorithm 2)

The plain algorithm is the simplest among the three encoding strategies considered in this manuscript. As it makes no effort to avoid redundant operations, it may at first glance seem generally inferior to the more sophisticated overlap and lookup algorithms (Algorithms 3 and 4). Quite the contrary, the plain algorithm may actually be preferable when numerical scripting languages like GNU Octave, Matlab or NumPy/Python shall be used to encode ordinal patterns. The reason is that, by contrast with the recursive Algorithms 3 and 4, the plain algorithm allows for a vectorised implementation that avoids loops.

Compared to pre-compiled languages like C, scripting languages are relatively slow at iterating loops. This clearly manifests if we implement the plain algorithm in a straightforward manner, which then requires three levels of nested loops. See the encode_plain functions in the supplementary files encode_plain.m and ordpat.py, as well as the ordpat_encode_plain function in ordpat.c, respectively. As shown in Table 4, the run-time efficiency varies by orders of magnitude across different execution environments.

Those differences are due to the iterative nature of the plain algorithm, which forces the Octave and Python language interpreters to translate the same sequence of instructions over and over again, for each and every loop iteration. Consistently, the Matlab just-in-time compiler performs better, but is in turn outperformed by the machine code of the fully-optimising C compiler. To mitigate the performance penalty inherent to numerical scripting languages, a programming technique known as vectorisation can be applied in many cases. In a nutshell, vectorisation is about avoiding element-wise operations in favour of high-level instructions acting on blocks of data, like vectors (hence the name), matrices, and tensors. Vectorising the plain algorithm is a bit tricky, but the performance gain is well worth the effort. The approach is best explained by means of a practical example. We will be using Matlab code here, but the concepts translate to other programming environments as well. Recall from Equation (11) that the map

(23) $$\left(x_{1},x_{2},\ldots ,x_{m}\right)\mapsto \sum_{i=1}^{m-1}\left(\left(m-i\right)!\;\sum_{j=i+1}^{m}\left[x_{i}>x_{j}\right]\right)$$

is the basis of the plain algorithm, and yields the ordinal pattern of the *m*-tuple $\left(x_{1},x_{2},\ldots ,x_{m}\right)$ in its numerical representation. Now consider that, for any fixed pattern order *m*, the result of this function can be rewritten without relying on summation signs. Assuming the order m=5, for example, the mathematical expression

24×x1>x2+x1>x3+x1>x4+x1>x5+6×x2>x2+x2>x3+x2>x4+x2>x5+2×x3>x2+x3>x3+x3>x4+x3>x5+1×x4>x2+x4>x3+x4>x4+x4>x5

is admittedly more tedious, but arithmetically equivalent to the compact formulation in Equation (23). The point here is that this expression can directly be translated into a single Matlab instruction, namely

       24 * ( (x1 > x2) + (x1 > x3) + (x1 > x4) + (x1 > x5) ) ...

               +   6 * (             (x2 > x3) + (x2 > x4) + (x2 > x5) ) ...  

               +   2 * (                         (x3 > x4) + (x3 > x5) ) ... 

               +   1 * (                                     (x4 > x5) );   

Let us assume that the time series to be analysed is represented by a N×1 vector input on the Matlab workspace. Obviously, if we initialise the variables

x1=input(1);x2=input(2);x3=input(3);x4=input(4);x5=input(5);

and call the above instruction, we obtain the ordinal pattern of the vector input(1:5) in its numerical representation. This is an optimisation technique called loop unrolling. Furthermore, consider that, in numerical scripting languages, most basic operations are not limited to scalar values, but can in principle handle arrays of arbitrary dimension as their operands. If we thus set x1, ..., x5 to the delay vectors

x1 = input(1:end-4);

x2 = input(2:end-3);

x3 = input(3:end-2);

x4 = input(4:end-1);

x5 = input(5:end-0);

instead, we can obtain from input its full sequence of ordinal patterns of order m=5 and lag τ=1 by calling a single Matlab instruction. Arbitrary time lags τ⩾1 can in turn be realised by using the generalised delay vectors

x1 = input(1 + 0*lag : end - 4*lag);

x2 = input(1 + 1*lag : end - 3*lag);

x3 = input(1 + 2*lag : end - 2*lag);

x4 = input(1 + 3*lag : end - 1*lag);

x5 = input(1 + 4*lag : end - 0*lag);

in conjunction with the exact same Matlab expression. As a side note, the operation is also applicable to multidimensional data structures like matrices and tensors, such that multivariate time series can as well be encoded by means of a single invocation.

The downside of this approach is that each pattern order *m* requires a dedicated piece of code. When working with low pattern orders, implementation by hand may be acceptable because it still yields comprehensible code—as is demonstrated by the symbolise.m function provided in the supplements of [40]. For higher pattern orders, though, another convenient feature offered by scripting languages should rather be utilised. GNU Octave, Matlab and NumPy/Python all support self-modifying code, which is source code that can dynamically modify its own sequence of instructions at run-time. Supporting languages provide built-in functions like eval or exec for this purpose, which take a string as their input argument and hand it over to the execution engine for evaluation. Such language facilities can neatly be utilised to obtain an efficient, universal implementation of the plain algorithm: we just have to write a function that dynamically creates the appropriate vectorised code for a given pattern order *m* and time lag τ, then executes it. Consider the functions encode_vectorised in the supplementary file encode_vectorised.m and ordpat.py for reference. As can be seen from Table 5, the optimisation yields a tremendous increase in run-time efficiency.

For any of the numerical computation environments considered, using a vectorised version of the plain algorithm allows for encoding millions of ordinal patterns in milliseconds, without having to rely on pre-compiled external libraries. Most noteworthy, the vectorised NumPy/Python implementation actually outperformed the pre-compiled C code in the majority of cases. This hints at the amount of sophistication put into the development of the free and open source NumPy/Python framework.

### 5.6. The Overlap Algorithm (Algorithm 3)

**Figure 3:**
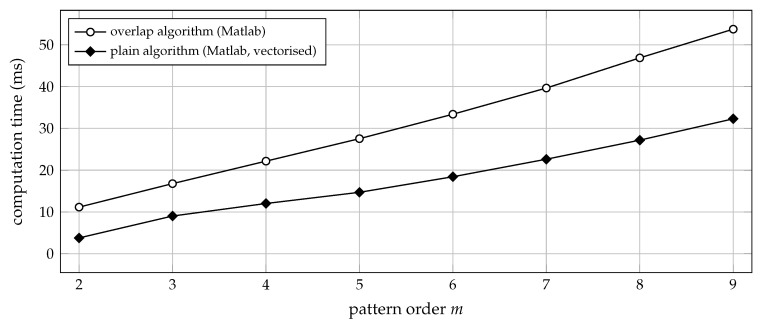
Computation time (median of 20 trials) for transforming $3.6\times 10^{5}$ samples of uniform white noise into a sequence of ordinal patterns of order *m*. The lag was set to a constant τ=1, and the Matlab functions encode_vectorised and encode_overlap from the supplementary file encode_vectorised.m and encode_overlap.m were used for the simulation.

The prerequisite for vectorising an algorithm is that all input data be available in advance, such that they can be passed to the software in parallel. Therefore, and by contrast with the plain algorithm considered above, recursive solutions like the overlap algorithm (Algorithm 3) cannot be fully vectorised, but inevitably require some sort of iteration. Due to the reasons given in Section 5.5, implementing the overlap algorithm in a numerical scripting language thus defeats its very purpose, which is the efficient evaluation of Equation (15). This can be demonstrated by benchmarking a Matlab implementation of the overlap algorithm against a vectorised Matlab implementation of the plain algorithm (Algorithm 2). The code for both implementations is provided in the supplements. While the overlap algorithm should be superior in theory, a vectorised implementation of the plain algorithm can actually be faster under practical conditions, as can be observed in Figure 3.

**Figure 4:**
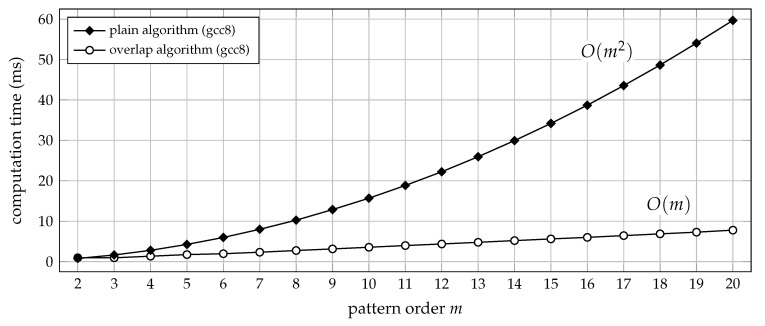
Computation time (median of 20 trials) for transforming $3.6\times 10^{5}$ samples of uniform white noise into a sequence of ordinal patterns of order *m*. The time lag was set to τ=1, and the C functions ordpat_encode_plain and ordpat_encode_overlap from the supplementary file ordpat.c were used for the simulation. The run-time complexity of the plain algorithm is $O(m^{2})$, whereas the overlap algorithm generally scales at O(m). For m=2, there is no advantage over the plain algorithm: all order relations are then disjoint, such that no overlap can be exploited (see Section 2.3).

To benefit from the overlap algorithm in terms of efficiency, it is therefore highly advisable to use a compiled programming language instead. In the following, we shall exclusively be concerned with implementing the overlap algorithm in the C programming language. For pattern orders up to m=20, the pseudocode of Algorithm 3 can directly be translated into C code (assuming 64-bit integer support on the targeted platform). Admittedly, a few minor tweaks are still possible, but those are easily understood from the reference implementation, that is, from the ordpat_encode_overlap function provided in the supplementary ordpat.c file. Typical run-time performances achieved by C implementations of the plain versus the overlap algorithm are depicted in Figure 4.

The aforementioned limitation to pattern orders m⩽20 results from the relation $20!<2^{64}<21!$, which implies that patterns beyond m=20 cannot be represented by 64-bit integers (see Section 5.2). Reconsidering the pseudocode of Algorithm 3, it is easily confirmed that the only instruction actually affected by this limitation is

(24) $$n_{t}\leftarrow \left(m-j\right)\left(n_{t}+r_{i,\;j}\right)$$

in line 27 of the listing: if the variable $n_{t}$ is limited to 64 bits of accuracy, it will eventually overflow for pattern orders m>20. Fortunately, it merely takes

**Figure 5:**
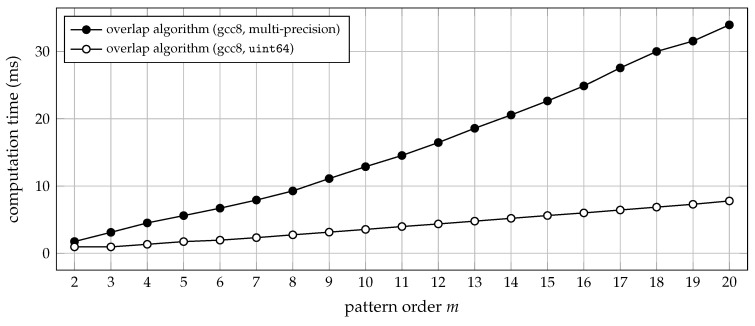
Computation time (median of 20 trials) for transforming $3.6\times 10^{5}$ samples of uniformly distributed white noise into a sequence of ordinal patterns of pattern order *m*, using the constant time lag τ=1. The C functions ordpat_encode_overlap and ordpat_encode_overlap_mp from the supplementary file ordpat.c were used for the simulation. The arbitrary-precision arithmetic used in the ordpat_encode_overlap_mp function increases the overall run-time complexity, and the timing is less stable than for strictly hardware-based arithmetic operations.

**Figure 6:**
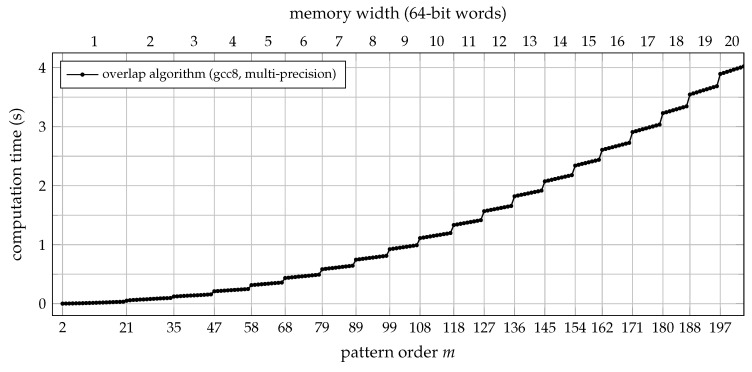
Computation time (median of 20 trials) for transforming $3.6\times 10^{5}$ samples of uniformly distributed white noise into a sequence of ordinal patterns of order *m*. The time lag was set to τ=1, and the C function ordpat_encode_overlap_mp (an arbitrary-precision implementation of the overlap algorithm) from the supplementary file ordpat.c was used for the simulation. The memory required per pattern is growing with *m* in a stepwise manner, increasing by one 64-bit word at each vertical grid line. The computational cost in turn rises linearly with the number of memory words to be iterated for each pattern, which shows as distinct jumps in the graph. Independent of that, the run-time complexity also increases linearly with the pattern order *m* as such. Both effects combined explain the parabolic envelope of the curve depicted.

1. an arbitrary-precision integer representation for the variable $n_{t}$,
2. a function that adds a non-negative integer to $n_{t}$, and
3. a function that multiplies $n_{t}$ with a non-negative integer

to overcome this upper boundary. Although books have been written about the details of arbitrary precision arithmetic [41,42], a simplistic approach will fully satisfy the current application. For an arbitrary pattern of order *m*, its maximum bit width is known in advance, and amounts to $\log_{2}m!$ bit. To ease subsequent analyses, it is advisable to keep the width constant across all patterns of a given order, such that we do not have to be concerned with dynamic memory reallocation. Any operation applied to an arbitrary-precision variable $n_{t}$ will then result in a fixed-length sequence of machine instructions, each acting on a fraction of the overall data. To minimise the instruction count, it thus makes sense to allocate an integer multiple of the machine word size per pattern. On contemporary hardware platforms, a total of

(25) $$d=\left\lceil \log_{2}\left(m!\right)/64\right\rceil$$

consecutive 64-bit words should be used for each ordinal pattern. Given a sequence of *N* ordinal patterns of order *m*, its resulting in-memory representation is then a 64-bit unsigned integer array comprised of d×N elements. The addition and multiplication functions required for the evaluation of Equation (24) should ideally act “in-place” on the array-valued operand $n_{t}$, while their respective second operands can safely be restricted to 32-bit unsigned integers. This is based on the conjecture that $m<2^{32}$ will not constitute a limitation in practice. The pair of arithmetical functions then boil down to a straightforward addition-with-carry, as well as a schoolbook multiplication approach. For reference, see the functions add_mp and multiply_mp, as well as the resulting multi-precision implementation of the overlap algorithm called ordpat_encode_overlap_mp (all to be found in the supplementary file ordpat.c). As is to be expected, the multi-precision approach introduces a certain performance penalty when compared to the standard implementation. This overhead is depicted in Figure 5 for the range of pattern orders supported by both variants. The run-time behaviour of ordpat_encode_overlap_mp for a wider range of pattern orders is in turn visualised in Figure 6.

As can be seen from Figure 5 and Figure 6, some of the run-time efficiency of the overlap algorithm has to be traded off to allow for pattern orders m>20, which require multi-precision (and thus, multi-iteration) integer arithmetic. Although the overall computational complexity then scales with $O(m^{2})$ again, the absolute run-time of the approach is still acceptable: encoding a one-hour sequence of data sampled at 100Hz will take less than one second of processing time for orders as high as m=100, for which an inconceivable number of $100!\approx 9.3\times 10^{157}$ distinct ordinal patterns do formally exist.

### 5.7. The Lookup Algorithm (Algorithm 4)

In analogy with the overlap algorithm (Algorithm 3) discussed in Section 5.6, the lookup algorithm (Algorithm 4) cannot be fully vectorised due to its recursive nature. Regarding run-time efficiency, numerical scripting languages are thus at a considerable disadvantage compared to compiled programming languages. We therefore implemented the lookup algorithm in the C programming language to enable meaningful comparison with Algorithm 3 (see the ordpat_encode_lookup function provided in the supplementary ordpat.c file). Still, native implementations for numerical scripting languages are provided in the supplements for the sake of completeness. Those are meant to allow the reader a quick confirmation of the aforementioned performance drop.

In theory, the lookup algorithm (Algorithm 4) is computationally more efficient than the overlap algorithm (Algorithm 3). For each pattern to be encoded, the overlap algorithm has to calculate an integer representation n∈{0,1,…,m!−1} from a tuple of inversion counts $\left(r_{1},r_{2},\ldots ,r_{m}\right)$, whereas the lookup algorithm can fetch the result of this operation from memory. This reduces the number of computational operations per ordinal pattern (see Table 2).

**Figure 7:**
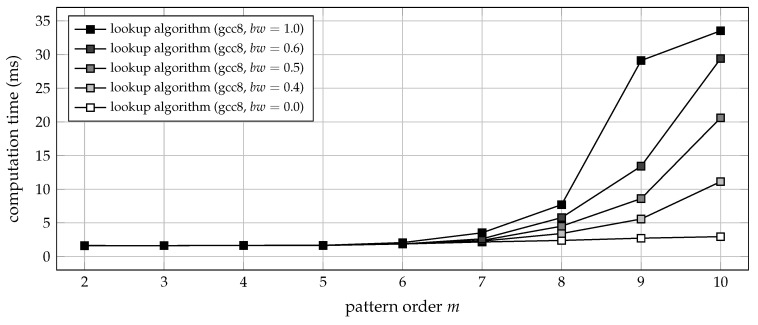
Computation time (median of 20 trials) for transforming $3.6\times 10^{5}$ samples of uniform white noise, low-pass filtered to various relative bandwidths bw, into sequences of ordinal patterns of order *m*. The time lag was set to a constant τ=1, and the C functions ordpat_encode_lookup and ordpat_encode_overlap from the supplementary file ordpat.c were used for the simulation. The time required for loading lookup tables from mass storage into main memory was not taken into account. Filtering to bw=0.0 results in an all-zero input signal, whereas bw=1.0 results in white noise. The computation time increases not only with the pattern order *m*, but also with the ordinal complexity of the input signal.

In practice, however, the overall run-time of a piece of software is not exclusively determined by the number of operations it performs, but also depends on memory requirements and memory access patterns. In this regard, and as described in Section 4.3, Algorithm 4 requires a lookup table of m!×m elements, each holding the numerical representation of a particular ordinal pattern of order *m*. Thus, the size of the lookup table increases rapidly with the pattern order: conservatively assuming $\log_{2}m!$ bit of storage space per pattern, the table size exceeds a gigabyte for m=11, and occupies more than five terabytes of memory for the order m=14. Therefore, memory access times quickly become prohibitive as the pattern order increases. For this reason, Unakafova and Keller stated that the applicability of their algorithm be limited to the pattern orders most commonly used [21], and provided precomputed lookup tables for m∈{2,3,…,9}. Nevertheless, the principal problem of memory access times still persists for low pattern orders. In cases where the lookup table is too large to entirely reside in the processor’s internal cache, the overall run-time efficiency of Algorithm 4 strongly depends on the nature of the input data. Time series of high ordinal complexity will then result in frequent cache misses. In other words: if the time series contains many different ordinal patterns, the processor will frequently have to reload different parts of the lookup table from main memory into cache, which stalls the processor and thus slows down the encoding process. This circumstance can be demonstrated by feeding low-pass filtered noise of increasing bandwidth to the lookup algorithm, which yields results as presented in Figure 7.

**Figure 8:**
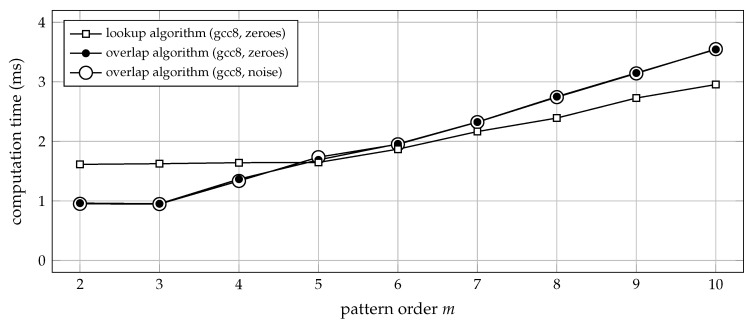
Computation time (median of 20 trials) for transforming $3.6\times 10^{5}$ samples of data into sequences of ordinal patterns of order *m*. Either zeroes or uniform white noise were used as input data. The time lag was set to a constant τ=1, and the C functions ordpat_encode_lookup and ordpat_encode_overlap from the supplementary file ordpat.c were used for the simulation. The time required for loading lookup tables from mass storage into main memory was not taken into account. For an all-zero input signal, no cache contention will occur, and the lookup algorithm can outperform the overlap algorithm as the pattern order *m* (and thus the computational workload for the overlap algorithm) increases.

It is therefore hard to draw a general conclusion on the run-time efficiency of the lookup algorithm. Suffice it to say that, for input data of low ordinal complexity, the lookup algorithm may outperform the overlap algorithm, as can be substantiated by using an all-zero time series as the test signal. In this idealised case, the algorithm will look up the exact same ordinal pattern again and again, and will therefore not run into cache contention issues, as is demonstrated in Figure 8.

Figure 8 also shows that the performance of the overlap algorithm is independent from the input data. It remains stable for both extreme cases: sequences of zeroes, as well as white noise. The benchmark presented in Figure 8 also conveys the impression that the overlap algorithm may be at an advantage for m∈{2,3,4} and any type of input signal. A possible explanation could be that addressing and accessing the lookup table in cache still imposes some constant delay, causing the processor to stall for the pattern orders with the lowest computational workload. However, considering that under the above conditions both algorithms achieve a data throughput of more than 1 GB per second, we did not study this effect any further: loading input data from mass storage will likely take a lot longer than the actual processing times considered here.

### 5.8. Sequence Length and Time Lag

**Figure 9:**
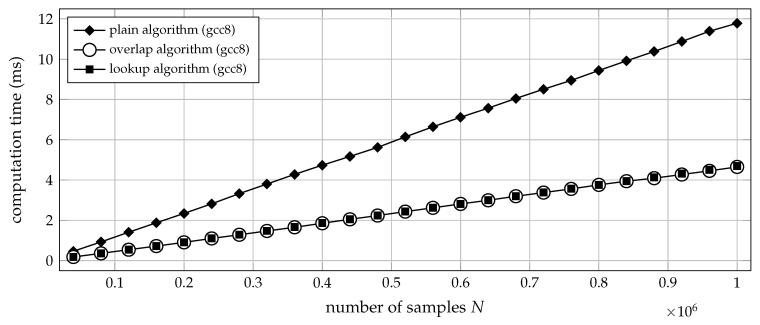
Computation time (median of 20 trials) for transforming *N* samples of uniform white noise into a sequence of ordinal patterns, using order m=5 and time lag τ=1. The respective C functions ordpat_encode_plain, ordpat_encode_overlap and ordpat_encode_lookup from the supplementary file ordpat.c were tested. The time required for loading lookup table data from mass storage into main memory was not taken into account. The order m=5 was selected so as to operate the ordpat_encode_lookup function at its sweet spot with regard to cache utilisation. In good approximation, the computation time then increases linearly with the sequence length *N* for all three algorithms.

In the previous simulations, signals of a fixed $N=3.6\times 10^{5}$ samples length have been processed, using the constant time lag τ=1 throughout. A remaining question therefore is how the plain, overlap and lookup algorithms (Algorithms 2–4) scale with regard to the length *N* of the input sequence, as well as the time lag τ under practical conditions. Fortunately, those aspects are qualitatively identical for all three algorithms, and their run-time behaviour is consistent with theoretical expectation: let aside the τ additional steps required to initialise the overlap and lookup algorithms, each of the algorithms is repeated once per ordinal pattern to be encoded, so doubling the amount of input data will coarsely double the computational effort. In addition, the data to be encoded are iterated in a linear manner. Therefore, neither inordinate cache misses nor incorrect branch prediction should pose a problem in theory. Simulation confirms that the run-time of all three algorithms scales linearly with the sequence length, as can be observed in Figure 9.

**Figure 10:**
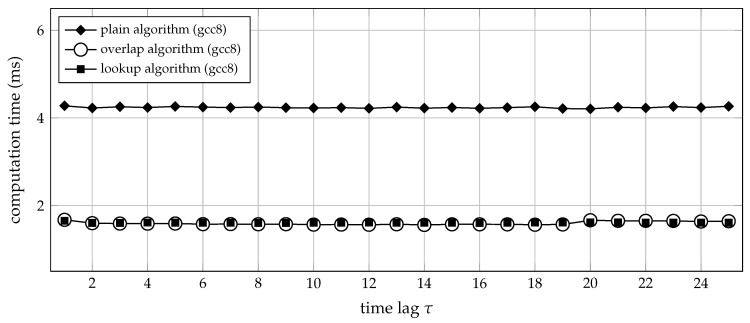
Computation time (median of 20 trials) for transforming $3.6\times 10^{5}$ samples of uniform white noise into a sequence of ordinal patterns, using the fixed order m=5 and increasing time lags τ. The respective C functions ordpat_encode_plain, ordpat_encode_overlap and ordpat_encode_lookup from the supplementary file ordpat.c were tested. The time required for loading lookup table data from mass storage into main memory was not taken into account. The order m=5 was selected so as to operate the ordpat_encode_lookup function at its sweet spot with regard to cache utilisation. The simulations did not reveal any noticeable dependency between the time lag τ and the computation time.

The relation between the time lag τ and the overall run-time is even simpler. With respect to computational effort, the time lag should not make any difference because, for all three algorithms, the value of τ is predominantly used to calculate memory addresses, where its absolute value cannot have any influence on the computational workload. On the other hand, τ determines the stride of the (otherwise linear) memory access pattern. Therefore, increasing the time lag could theoretically be detrimental to the cache performance. Under test conditions, however, the choice of τ had no noticeable influence on run-time efficiency: consider the measurements depicted in Figure 10.

## 6. Conclusions

Three different algorithms were discussed that all analyse the ordinal patterns (see Definition 1) of a given time series, and encode them in a computationally advantageous way, such that the ordinal patterns $\left\{\Pi_{1},\Pi_{2},\ldots ,\Pi_{m!}\right\}$ of order *m* are compactly represented by the set of non-negative integers {0,1,…,m!−1} in a one-to-one manner. The theoretical foundations for this encoding were adopted from the Lehmer code [33], a classical approach in computational combinatorics (see Section 3).

### 6.1. Picking the Right Tool for the Job

From a theoretical perspective, the plain algorithm (Algorithm 2) has the highest computational complexity, followed by the overlap algorithm (Algorithm 3), and in turn followed by the lookup algorithm (Algorithm 4), which is the least computationally complex among the three (see Section 5.1). In practice, however, the algorithms presented are complementary with regard to their scope of application, and each can be worth considering.

Being fully vectorisable, the plain algorithm (Algorithm 2) is a particularly good choice for computational environments like Matlab, GNU Octave or NumPy/Python, and its efficiency should suffice most standard applications of ordinal pattern analysis (see Table 5).

By contrast, the overlap algorithm (Algorithm 3) constitutes a general-purpose solution, providing high data throughput over a wide range of pattern orders *m*, while only requiring a small amount of extra memory. To achieve suitable run-time performance, it needs to be implemented in a compiled programming language, though. This is not an actual limitation in practice because virtually any high-level scripting language can link against pre-compiled library functions. Under this paradigm of execution, the overlap algorithm clearly outperforms the plain algorithm. Implementing the overlap algorithm in the C programming language is straightforward, and provides plenty of opportunity for platform-specific optimisation: as demonstrated in Section 5.6, arbitrary-precision arithmetic can easily be incorporated to enable pattern orders m>20, and (although not considered in this manuscript) single-instruction-multiple-data (SIMD) processing could be employed to further boost the run-time performance on supporting architectures. With regard to real-time applications running on specialised embedded systems, it may be worth mentioning that the algorithm does not depend on floating-point arithmetic, and merely uses a few extra bytes of working memory on top of its input/output buffers.

As with the overlap algorithm, the lookup algorithm (Algorithm 4) should ideally be implemented in a compiled programming language to maximise its performance. By matter of principle, it has a narrower scope of application, though. Depending on a lookup table of m!×m elements, its memory requirements currently limit the algorithm to pattern orders m∈{2,3,…,10}. For the same reason, its run-time performance varies with the nature of the input data (see Figure 7). When analysing time series of comparatively low ordinal complexity, the lookup algorithm may outperform the overlap algorithm. On the other hand, time series of higher ordinal complexity will result in frequent cache misses, and may lead to a substantial drop in the overall run-time. Thus, if performance is critically important, both algorithms should be tested for the particular kind of data to be analysed.

### 6.2. Final Remarks

The general aim of the present article is to improve the run-time performance of known methods in ordinal pattern analysis, and to foster the development of new applications, so far hindered by computational limitations. To that end, the publication is supplemented by a cross-platform software library that supports various programming languages commonly used in scientific computing, namely NumPy/Python, GNU Octave, Matlab, as well as the C programming language. The library includes reference implementations for all algorithmic variants considered here, and is provided under the permissive terms of a 3-clause Berkeley Software Distribution (BSD) license.

Mapping time series onto sequences of ordinal patterns is essential to the methodology, but constitutes only the first step of ordinal pattern analysis. Other aspects often include the estimation of (possibly multi-dimensional) probability masses. As soon as the pattern order *m* increases beyond a certain point, those can pose a computational challenge in their own right. Thus, a follow-up article discussing such matters is currently under preparation.

Last but not least, it has to be stated clearly that the present work is exclusively concerned with computational feasibility as such, and in no way with the actual relevance of high pattern orders. Most prominently, the part on multi-precision arithmetic in Section 5.6 was written in the hope that it will be useful to researchers further exploring the possibilities of ordinal pattern analysis. That being said, the authors do not endorse the extension of well-established analysis methods to unreasonably high pattern orders: for a fixed sequence length *N*, not only computational efficiency, but also statistical validity may vanish quite rapidly as the pattern order *m* increases.

## Figures and Tables

**Table 1:** The ranks $\left(\lambda_{1},\lambda_{2},\lambda_{3},\lambda_{4}\right)$, right inversion counts $\left(r_{1},r_{2},r_{3}\right)$, and numerical representations *n* for the m!=24 ordinal patterns of order m=4.

Ranks				Inversions			Code	Ranks				Inversions			Code
$\lambda_{1}$	$\lambda_{2}$	$\lambda_{3}$	$\lambda_{4}$	$r_{1}$	$r_{2}$	$r_{3}$	*n*	$\lambda_{1}$	$\lambda_{2}$	$\lambda_{3}$	$\lambda_{4}$	$r_{1}$	$r_{2}$	$r_{3}$	*n*
1	2	3	4	0	0	0	0	3	1	2	4	2	0	0	12
1	2	4	3	0	0	1	1	3	1	4	2	2	0	1	13
1	3	2	4	0	1	0	2	3	2	1	4	2	1	0	14
1	3	4	2	0	1	1	3	3	2	4	1	2	1	1	15
1	4	2	3	0	2	0	4	3	4	1	2	2	2	0	16
1	4	3	2	0	2	1	5	3	4	2	1	2	2	1	17
2	1	3	4	1	0	0	6	4	1	2	3	3	0	0	18
2	1	4	3	1	0	1	7	4	1	3	2	3	0	1	19
2	3	1	4	1	1	0	8	4	2	1	3	3	1	0	20
2	3	4	1	1	1	1	9	4	2	3	1	3	1	1	21
2	4	1	3	1	2	0	10	4	3	1	2	3	2	0	22
2	4	3	1	1	2	1	11	4	3	2	1	3	2	1	23

**Table 2:** Number of operations required to encode a single ordinal pattern of order *m*, using either the plain algorithm (Algorithm 2), the overlap algorithm (Algorithm 3), or the lookup algorithm (Algorithm 4). Early initialisation operations have not been considered.

Algorithm	Add	Multiply	Compare	Increment	Assign	Modulo	Total
plain	$\frac{m^{2}+3m-2}{2}$	m−1	$m^{2}-1$	$\frac{m^{2}+m-2}{2}$	$\frac{m^{2}+3m}{2}$	0	$\frac{5m^{2}+9m-8}{2}$
overlap	9m−8	6m−6	2m−2	*m*	2m	1	20m−15
lookup [21]	6m−3	2m−1	2m−2	m−1	m+3	0	12m−4

**Table 3:** Maximum pattern orders representable by standard integer and floating point data types.

Data Type	Significant Bits	Maximum Order
	$w_{b}$	*m*
uint8	8	5
uint16	16	8
uint32	32	12
uint64	64	20
binary32 (single)	24	10
binary64 (double)	53	18

**Table 4:** Computation time (median of 20 trials) for turning $3.6\times 10^{5}$ samples of uniform white noise into a sequence of ordinal patterns of order *m*, using the time lag τ=1. Straightforward iterative implementations of Algorithm 2 were tested in various computation environments.

Order	Computation Time (ms)			
*m*	GNU Octave	NumPy/Python	Matlab	C
2	$7.9\times 10^{3}$	$2.4\times 10^{3}$	$1.1\times 10^{1}$	$7.8\times 10^{-1}$
3	$2.0\times 10^{4}$	$5.7\times 10^{3}$	$2.2\times 10^{1}$	$1.6\times 10^{0}$
4	$3.6\times 10^{4}$	$1.0\times 10^{4}$	$3.5\times 10^{1}$	$2.8\times 10^{0}$
5	$5.6\times 10^{4}$	$1.5\times 10^{4}$	$5.5\times 10^{1}$	$4.3\times 10^{0}$
6	$8.1\times 10^{4}$	$2.1\times 10^{4}$	$8.5\times 10^{1}$	$6.0\times 10^{0}$
7	$1.1\times 10^{5}$	$2.9\times 10^{4}$	$1.2\times 10^{2}$	$8.0\times 10^{0}$
8	$1.4\times 10^{5}$	$3.7\times 10^{4}$	$1.6\times 10^{2}$	$1.0\times 10^{1}$
9	$1.8\times 10^{5}$	$4.6\times 10^{4}$	$2.0\times 10^{2}$	$1.3\times 10^{1}$

**Table 5:** Computation time (median of 20 trials) for turning $3.6\times 10^{5}$ samples of uniform white noise into a sequence of ordinal patterns of order *m*, using the time lag τ=1. Vectorised implementations of Algorithm 2 were tested in various computation environments. The results of Table 4 (relating to iterative implementations of Algorithm 2) were replicated for ease of comparison.

Order	Computation Time (ms)						
*m*	GNU Octave		NumPy/Python		Matlab		C
	loops	vectors	loops	vectors	loops	vectors	loops
2	$7.9\times 10^{3}$	$1.5\times 10^{0}$	$2.4\times 10^{3}$	$7.9\times 10^{-1}$	$1.1\times 10^{1}$	$3.8\times 10^{0}$	$7.8\times 10^{-1}$
3	$2.0\times 10^{4}$	$6.5\times 10^{0}$	$5.7\times 10^{3}$	$1.3\times 10^{0}$	$2.2\times 10^{1}$	$9.0\times 10^{0}$	$1.6\times 10^{0}$
4	$3.6\times 10^{4}$	$1.4\times 10^{1}$	$1.0\times 10^{4}$	$2.0\times 10^{0}$	$3.5\times 10^{1}$	$1.2\times 10^{1}$	$2.8\times 10^{0}$
5	$5.6\times 10^{4}$	$2.0\times 10^{1}$	$1.5\times 10^{4}$	$2.9\times 10^{0}$	$5.5\times 10^{1}$	$1.5\times 10^{1}$	$4.3\times 10^{0}$
6	$8.1\times 10^{4}$	$3.3\times 10^{1}$	$2.1\times 10^{4}$	$5.3\times 10^{0}$	$8.5\times 10^{1}$	$1.8\times 10^{1}$	$6.0\times 10^{0}$
7	$1.1\times 10^{5}$	$4.2\times 10^{1}$	$2.9\times 10^{4}$	$6.8\times 10^{0}$	$1.2\times 10^{2}$	$2.3\times 10^{1}$	$8.0\times 10^{0}$
8	$1.4\times 10^{5}$	$5.7\times 10^{1}$	$3.7\times 10^{4}$	$8.5\times 10^{0}$	$1.6\times 10^{2}$	$2.7\times 10^{1}$	$1.0\times 10^{1}$
9	$1.8\times 10^{5}$	$6.8\times 10^{1}$	$4.6\times 10^{4}$	$1.3\times 10^{1}$	$2.0\times 10^{2}$	$3.2\times 10^{1}$	$1.3\times 10^{1}$

## Supplementary Materials

The supplementary code is available online at https://www.mdpi.com/1099-4300/21/10/1023/s1, and will be developed further at https://github.com/seb-berger/libordpat.

## References

[1] Bandt C., Pompe B. (2002). Permutation Entropy: A Natural Complexity Measure for Time Series. Phys. Rev. Lett..

[2] Shannon C.E. (1948). A Mathematical Theory of Communication. Bell Syst. Tech. J..

[3] Shannon C.E. (1948). A Mathematical Theory of Communication. Bell Syst. Tech. J..

[4] Zanin M., Zunino L., Rosso O.A., Papo D. (2012). Permutation Entropy and Its Main Biomedical and Econophysics Applications: A Review. Entropy.

[5] Amigó J.M., Keller K., Kurths J. (2013). Recent Progress in Symbolic Dynamics and Permutation Complexity. Eur. Phys. J. Spec. Top..

[6] Amigó J.M., Keller K., Unakafova V.A. (2014). Ordinal symbolic analysis and its application to biomedical recordings. Philos. Trans. R. Soc. A Math. Phys. Eng. Sci..

[7] Fadlallah B., Chen B., Keil A., Príncipe J. (2013). Weighted-permutation entropy: A complexity measure for time series incorporating amplitude information. Phys. Rev. E.

[8] Li D., Li X., Liang Z., Voss L.J., Sleigh J.W. (2010). Multiscale permutation entropy analysis of EEG recordings during sevoflurane anesthesia. J. Neural Eng..

[9] Morabito F.C., Labate D., La Foresta F., Bramanti A., Morabito G., Palamara I. (2012). Multivariate Multi-Scale Permutation Entropy for Complexity Analysis of Alzheimer’s Disease EEG. Entropy.

[10] Azami H., Escudero J. (2016). Amplitude-aware permutation entropy: Illustration in spike detection and signal segmentation. Comput. Methods Programs Biomed..

[11] Unakafov A.M., Keller K. (2014). Conditional entropy of ordinal patterns. Phys. D Nonlinear Phenom..

[12] King J.R., Sitt J.D., Faugeras F., Rohaut B., El Karoui I., Cohen L., Naccache L., Dehaene S. (2013). Information sharing in the brain indexes consciousness in noncommunicative patients. Curr. Biol..

[13] Schreiber T. (2000). Measuring Information Transfer. Phys. Rev. Lett..

[14] Staniek M., Lehnertz K. (2008). Symbolic Transfer Entropy. Phys. Rev. Lett..

[15] Groth A. (2005). Visualization of coupling in time series by order recurrence plots. Phys. Rev. E.

[16] Bandt C., Shiha F. (2007). Order patterns in time series. J. Time Ser. Anal..

[17] Keller K., Sinn M., Emonds J. (2007). Time Series From the Ordinal Viewpoint. Stochastics Dyn..

[18] Amigó J.M. (2010). Permutation Complexity in Dynamical Systems.

[19] Cao Y., Tung W.w., Gao J.B., Protopopescu V.A., Hively L.M. (2004). Detecting dynamical changes in time series using the permutation entropy. Phys. Rev. E.

[20] Frank B., Pompe B., Schneider U., Hoyer D. (2006). Permutation entropy improves fetal behavioural state classification based on heart rate analysis from biomagnetic recordings in near term fetuses. Med. Biol. Eng. Comput..

[21] Unakafova V.A., Keller K. (2013). Efficiently Measuring Complexity on the Basis of Real-World Data. Entropy.

[22] Zunino L., Olivares F., Scholkmann F., Rosso O.A. (2017). Permutation entropy based time series analysis: Equalities in the input signal can lead to false conclusions. Phys. Lett. A.

[23] Dickten H., Lehnertz K. (2014). Identifying delayed directional couplings with symbolic transfer entropy. Phys. Rev. E.

[24] Unakafov A.M., Keller K. (2018). Change-Point Detection Using the Conditional Entropy of Ordinal Patterns. Entropy.

[25] Kaiser A., Schreiber T. (2002). Information transfer in continuous processes. Phys. D Nonlinear Phenom..

[26] Staniek M., Lehnertz K. (2007). Parameter selection for permutation entropy measurements. Int. J. Bifurc. Chaos.

[27] Amigó J.M., Kocarev L., Szczepanski J. (2006). Order patterns and chaos. Phys. Lett. A.

[28] Amigó J.M., Zambrano S., Sanjuán M.A.F. (2007). True and false forbidden patterns in deterministic and random dynamics. Europhys. Lett..

[29] Riedl M., Müller A., Wessel N. (2013). Practical considerations of permutation entropy. Eur. Phys. J. Spec. Top..

[30] Iverson K.E. (1962). A Programming Language.

[31] Knuth D.E. (1992). Two Notes on Notation. Am. Math. Mon..

[32] Laisant C.A. (1888). Sur la numération factorielle, application aux permutations. Bull. la Société Mathématique Fr..

[33] Lehmer D.H., Bellman R., Hall M. (1960). Teaching combinatorial tricks to a computer. Proceedings of Symposia in Applied Mathematics.

[34] Eaton J.W. (2012). GNU Octave and reproducible research. J. Process Control.

[35] Oliphant T.E. (2007). Python for Scientific Computing. Comput. Sci. Eng..

[36] (1985). IEEE 754-1985: IEEE Standard for Binary Floating-Point Arithmetic.

[37] (2008). IEEE 754-2008: IEEE Standard for Floating-Point Arithmetic.

[38] Goldberg D. (1991). What every computer scientist should know about floating-point arithmetic. ACM Comput. Surv..

[39] Marsaglia G. (2003). Xorshift RNGs. J. Stat. Softw..

[40] Berger S., Schneider G., Kochs E.F., Jordan D. (2017). Permutation Entropy: Too Complex a Measure for EEG Time Series?. Entropy.

[41] St. Denis T., Rose G. (2006). BigNum Math: Implementing Cryptographic Multiple Precision Arithmetic.

[42] Brent R.P., Zimmermann P. (2010). Modern Computer Arithmetic.

